# Mobile Phone and Tablet Apps to Support Young People’s Management of Their Physical Long-Term Conditions: A Systematic Review Protocol

**DOI:** 10.2196/resprot.4159

**Published:** 2015-04-07

**Authors:** Rabiya Majeed-Ariss, Andrew G Hall, Janet McDonagh, Deborah Fallon, Veronica Swallow

**Affiliations:** ^1^School of Psychological SciencesUniversity of ManchesterManchesterUnited Kingdom; ^2^School of Nursing, Midwifery and Social WorkFaculty of Medicine and Human SciencesUniversity of ManchesterManchesterUnited Kingdom; ^3^Institute of Child Health Birmingham Children's Hospital NHS Foundation TrustUniversity of BirminghamBirminghamUnited Kingdom; ^4^School of HealthcareFaculty of Medicine and HealthUniversity of LeedsLeedsUnited Kingdom

**Keywords:** mobile app, mobile phone, protocol, smartphone, tablets, young people, long-term conditions, chronic conditions, management, systematic review

## Abstract

**Background:**

The prevalence of long-term or chronic conditions that limit activity and reduce quality of life in young people aged 10-24 years is rising. This group has distinct health care needs and requires tailored support strategies to facilitate increasing personal responsibility for the management of their condition wherever possible, as they mature. Mobile phone and tablet mobile technologies featuring software program apps are already well used by young people for social networking or gaming. They have also been utilized in health care to support personal condition management, using condition-specific and patient-tailored software. Such apps have much potential, and there is an emerging body of literature on their use in a health context making this review timely.

**Objective:**

The objective of this paper is to develop a systematic review protocol focused on identifying and assessing the effectiveness of mobile phone and tablet apps that support young people’s management of their chronic conditions.

**Methods:**

The search strategy will include a combination of standardized indexed search terms and free-text terms related to the key concepts of young people; long-term conditions and mobile technology. Peer-reviewed journal articles published from 2003 that meet the inclusion and exclusion criteria will be identified through searching the generated hits from 5 bibliographical databases. Two independent reviewers will screen the titles and abstracts to determine which articles focus on testing interventions identified as a mobile phone or tablet apps, and that have been designed and delivered to support the management of long-term conditions in young people aged 10-24 years. Data extraction and quality assessment tools will be used to facilitate consistent analysis and synthesis. It is anticipated that several studies will meet the selection criteria but that these are likely to be heterogeneous in terms of study design, reported outcomes, follow-up times, participants’ age, and health condition. Sub-group analyses will be undertaken and where possible meta-analyses will take place.

**Results:**

This review will synthesize available knowledge surrounding tablet and mobile phone apps that support management of long term physical health conditions in young people. The findings will be synthesized to determine which elements of the technologies were most effective for this population.

**Conclusions:**

This systematic review aims to synthesize existing literature in order to generate findings that will facilitate the development of an app intervention. The review will form the first phase of development and evaluation of a complex intervention as recommended by the United Kingdom Medical Research Council. The knowledge gained from the review will be verified in subsequent phases, which will include primary qualitative work with health professionals and young people with long term conditions as research participants. Young people living with long-term conditions will be involved as co-researchers and consumer advisors in all subsequent phases to develop and evaluate an app to support the management of long-term physical health conditions.

**Trial Registration:**

PROSPERO International prospective register of systematic reviews: CRD42014015418; http://www.crd.york.ac.uk/PROSPERO/display_record.asp?ID=CRD42014015418#.VRqCpTpnL8E (Archived by Webcite at http://www.webcitation.org/6XREcWqQY).

## Introduction

### Young People With Physical Long-Term Conditions

Globally, the pattern of illness in young people aged 10-24 years (hereafter referred to as young people) has shifted from acute episodes, to long-term or chronic conditions that will potentially affect them across the life course [1]. At least 12% of young people have a long-term or chronic condition, but the actual number with one or more conditions is unknown [2]. A long-term or chronic condition is defined as a health condition that, at the time of diagnosis, is predicted to last longer than 3 months [3] (hereafter described as a long-term condition). However, there is an increased prevalence of long-term conditions that are severe enough to limit activity and delay normal developmental milestones, thus reducing young people’s quality of life and that of their parents/carers and families [4].

Survival rates for this group have improved due to better screening, earlier detection, and improvements in the delivery of specialized care [4,5]. However, there is growing evidence to suggest that young people with long-term conditions have distinct health needs when compared to other groups [6]. Effective support from the health sector is, therefore, paramount, especially during the transition from pediatric to adult health services, and particularly if adult services are not designed specifically to meet the needs of young-people [7]. This process of health transition as young people grow up requires them to develop clinical skills and knowledge in order to ultimately take responsibility for and competently manage their personal health where appropriate [2,8,9].

Delivering safe and timely health care that is accessible and tailored to the individuals’ needs and preferences is a central feature of international health care strategies [6]. Additionally, government policies highlight the need for services to support self-care, for example, the UK Department of Health and Department for Education are working to support young people with complex health needs in making the transition to adulthood [10].

A recent systematic review of self-care support interventions for children and young people found that effective interventions included those that used the Internet and text messaging for delivery, although none of the reviewed studies were reported to use mobile phone or tablet apps [11]. However, contemporaneous reports indicate that utilizing modern mobile electronic technologies in health interventions for adults of all ages [12,13] and young people [14] may be a suitable way to address self, shared, or joint care in a manner that is resource efficient.

The potential of mobile technologies in this area is increasingly recognized as being significant. For example, health management behaviors can be integrated with other daily activities by technologies that are capable of tracking information whilst “on the go”. An emerging body of literature on the use of mobile technologies in a health context makes a systematic review timely, to collate and build on lessons learnt as well as prevent duplication of research effort.

In this current review, young people are defined as those aged 10-24 years who are undergoing key elements of development, particularly brain development, which continues until the early 20s [1,15-17]. As increasing numbers of young people with long-term conditions are transitioning to adult-centered care, significant declines in treatment adherence have been observed during the transition period [18]. Interventions to enhance medication adherence found that education interventions alone are insufficient to promote adherence, but adding behavioral interventions such as monitoring and goal setting, reinforcing medication taking with rewards, contingency contracting, problem solving, and linking medication taking with established routines may enhance outcomes [19,20]. However, the small treatment effects of recent adherence-promoting interventions reflect the methodological limitations of the included studies and the need to reexamine the delivery and mechanisms of adherence-promoting interventions. Therefore, this is arguably a crucial time for the rigorous development, evaluation and implementation of interventions that promote shared and self-management skills and knowledge, and for the promotion of health-promoting behaviors.

### Mobile Phone and Tablet Apps to Support Management of Long-Term Conditions

The new generation of inexpensive, powerful, hand-held computers (mobile electronic devices) were first described in 1987 [21]. While the potential of these devices for patients and clinicians to collect field data more easily and reliably was quickly identified, limitations in terms of expense, responsiveness, connectivity, and evidence of their effectiveness, has affected their integration into practice. A Cochrane review in 2009 of Interactive Health Communication Applications (computer-based, usually Web-based, information packages for patients that combine health information with social support, decision support, and/or behavior change support) concluded that the mode or site of delivery is not important but did not report any studies involving mobile phones or tablet apps [22]. However, mobile phones and tablets now form the new generation of mobile electronic devices, chiefly different to previous generations in that they are a consumer product as opposed to primarily a business product [23]. Mobile phones and tablets have the additional feature of extending their function with custom software programs called apps, which technologically, allow the development of condition specific and patient tailored software. Additionally, mobile phones and tablets are primarily communication devices; whereas traditional Web-based apps have as their main foci information provision and/or gatekeeping to wider social networks. Mobile technologies and mobile phones in particular are personal devices, adapted by the user to reflect their specific needs. This personal nature of mobile devices (as opposed to a desktop or laptop computers), as well as the technology underpinning mobile apps, allows for adaptive, responsive, confidential, and targeted channels of communication and alerts.

In a recent review of the effectiveness of mobile health technology-based health behavior change or disease management interventions for adults, only 6 of the 49 disease management interventions used apps and none of these involved young people with long-term conditions [13]. Another recent integrative review of mobile phone interventions for long-term health management of chronic disease in patients aged 18-73 years [24] concluded that there are limited mobile apps available and recommends that more be developed. A review of the top 500 medical apps in the Italian health care android market showed that the majority were designed for health care professionals [25]. Since the potential of mobile technologies in health care is significant, a rapidly growing body of literature is currently emerging on the use of apps to support patients’ management of long-term conditions and a review of the evidence is timely.

In a recent commentary, Wu and Hommel [26] described current and potential technologies; such as text messaging, mobile phone apps, electronic monitors of adherence, and illness-specific medical devices to promote pediatric adherence to prescribed medical regimens. The reported uses included: delivering and collecting information, facilitating communication between patients and professionals, social networking, capturing real-time data, monitoring bodily functions, automated feedback, guidance and clinical alerts, and smart decision-making tools. However, despite the significant potential and increased use of these technologies, to our knowledge there has not been a synthesis of studies reporting on their effectiveness of these mobile technologies in the management of physical long-term health conditions in young people.

There are barriers to the use of mobile technologies by young people, including the disparity of access to mobile devices and the potential for habituation, suggesting that the use of IT to address health issues may be limited or even harmful to young people [27,28]. In addition, parents/carers play a significant part in promoting the development of young peoples’ self-management skills in long-term condition management [29], but parents may be less confident than young people in using technology [30]. Furthermore, in view of the relatively underdeveloped area of adolescent health services, it can be difficult for those health professionals who are unfamiliar with mobile phone and tablet apps to engage effectively with young people via these media [31,32]. Therefore, there is a need for well-designed trials of mobile phone and tablet app interventions that may be feasibly transferred into real-life settings and which involve parents, health professionals and young people in their development and evaluation.

Nevertheless mobile apps are widely acceptable to young people living in an increasingly technology-rich environment with good access to mobile phones and tablets in their day-to-day lives [33]. In the United Kingdom, children and young people aged 5-15 years are frequent users of mobile technologies: 62% of 12-15 year olds own a mobile phone, and the use of tablet computers by 5-15 year olds tripled between 2012 and 2013 with 42% using tablets in 2013 [23]. These trends are expected to continue and have the potential to engage young people in their personal health care. New technologies are emerging drivers in adolescent health with potential for both positive and negative impact [6].

In 2013, the UK National Health Service (NHS) Commissioning Board unveiled a library of NHS reviewed health apps [34]. Although this review focused on clinical safety rather than clinical effectiveness, it acknowledged that the computing capability contained within mobile technologies offers a legitimate platform for medical and public health practice. That said, the (IMS Health)-Institute of Healthcare Informatics [35], reported that the lack of evidence regarding the effectiveness of mobile apps acts as a barrier to physicians prescribing them. The IMS identified a pressing need for credible evidence of the value of health apps, which in many cases are being used without a thorough understanding of their associated risks and benefits or a rigorous, evidenced based approach to their development, evaluation, and validation [36]. Therefore, the review protocol presented in this paper will focus on assessing the effectiveness of mobile phone and tablet apps for young people’s management of long-term conditions.

##  Methods

### The Systematic Review

Mobile phone and tablet applications can be used in a host of ways to support the management of physical long-term conditions. Namely, these apps seek to define and refine the practices and procedures required for behavioral change; which in turn are anticipated to improve clinical and psychosocial outcomes.

Management of long-term physical health conditions involves 5 core skills: problem solving, decision making, resource utilization, forming patient-health care professional relationships, and taking action [37]. Apps can support these skills as well as knowledge development by providing and collecting information in a manner more accessible and convenient than that which existed previously, as well as having the additional advantage of interactivity.

For example, interventions delivered through a mobile phone or tablet app could include: an electronic diary which would serve as a medication or appointment reminder, a symptom monitor, a meaningful way of displaying clinical data to patients, educational materials tailored to individual patients’ needs and preferences, and/or a way of enabling patients to choose whether or not to share their data with health professional(s) for more meaningful consultations [38].

This systematic review will synthesize the evidence on all types of mobile phone and tablet apps that are used to support the management of physical long-term conditions in young people. Metaanalyses will be performed where possible. This systematic review will follow the methods described in the Cochrane Handbook for Systematic Reviews of Intervention [39], and be reported in compliance with the Preferred Reporting Items for Systematic Reviews and Meta-Analyses (PRISMA) guidelines [40].

### Search Strategy

Eligible studies will be identified through a comprehensive literature search of 5 bibliographical databases (MEDLINE, CINAHL, EMBASE, PsycINFO, and the Web of Science). The search strategy has been developed in consultation with an information scientist.

The search strategy uses a combination of standardized indexed search terms and free-text terms that relate to the three key concepts (young people; physical long-term conditions and mobile technology). The search includes British and North American terms and spellings. The search strategy was initially devised in MEDLINE and then adapted to the other databases (see Multimedia Appendix 1 for supporting information). The Web of Science does not employ any indexed search terms and the other databases did not employ them in a standardized fashion. Free-text terms have been used consistently throughout.

In addition to testing search sensitivity, those journals associated with the most retrieved citations will be hand-searched from 2009 to 2014. Supplementing the search with hand-searching decreases the likelihood of missing relevant studies. The production of any studies additional to those we have already identified from hand-searching will also allow us to comment on the rigour of the search strategy and the quality of indexing in the said bibliographic databases. This would be particularly useful in the relatively new domain of mobile technology. Also, due to the emerging nature of mobile technology, the search will include conference abstracts published in peer-reviewed journals, and authors will be contacted requesting additional related published or unpublished work.

### Screening and Selection Criteria

#### Overview

Two reviewers will independently screen all titles and abstracts retrieved by the search using a screening tool with study inclusion criteria as a prompt (see Textbox 1). Two reviewers will then independently screen full articles of the abstracts still included, using the same screening tool. Whenever disagreement in interpretation arises between the two reviewers, the rest of the team members will be asked to consult the relevant materials to enable a discussion until a consensus is reached, thereby minimizing bias in the interpretation of findings. Team meetings will be held regularly for the purpose of discussing articles and for discussion of complications or challenges.

#### Inclusion Criteria

Criteria for included studies are in Textbox 1. The Cochrane Collaboration states that a typical metaanalysis ought to exclude non-randomized controlled trials due to their greater bias. In spite of this, we have chosen to include studies of various designs to systematically collect a broad overview of the evidence. However, decisions on which studies to include in a metaanalysis will only be made after quality assessments are undertaken and risk of bias is ascertained.

**Summary of inclusion criteria.**

Population:

Young people aged 10-24 years old (WHO definition 2001 [1]) diagnosed with a long-term physical condition in any setting.

Intervention:

Any application for a mobile phone or tablet that can be considered a management intervention (or a component of an intervention) in terms of content and/or delivery. This judgment will be based on the 5 core management skills for long-term physical health conditions, as outlined by Lorig [34].

Comparisons:

Intervention versus usual care OR intervention variant versus intervention variant OR pre and post.

Outcomes:

Any physiological, attitudinal, behavioral or knowledge outcomes.

Study design:

Randomized controlled trial OR controlled clinical trial OR cohort analytic OR case-control OR cohort OR interrupted time series.

#### Exclusion Criteria

While international literature will be included, non-English language publications will be excluded from the review due to resource limitations. Interventions using mobile phone technology only in the context of delivering/receiving text messages or phone calls will also be excluded. Given the review focus, the technology context is considered key so we will apply a publication start date of 2003. This is the year when 3G networks (required by apps) were launched in the United Kingdom [41]. The nature of modern technology means that this date is arguably internationally applicable. Studies that focus on young people with mental health problems, learning disabilities, and cognitive impairment will be excluded from this review, although at a later date we will undertake a review of studies involving such young people to determine whether apps are effective in supporting their particular skill and knowledge development.

### Date Extraction

For every included study, two reviewers will extract relevant data independently. A tool based on the Data Extraction Template for Cochrane reviews [42] has been developed to facilitate consistent data extraction and prevent important information from being overlooked. This tool will be pilot tested, based on which, detailed instructions will be developed to make the process more objective. Any disagreements between reviewers will be resolved by discussion with the rest of the research team. The tool includes information regarding the study method (eg, study aim, intervention aim, study design, recruitment, participation criteria, ethics, funding, statistical methods used, and consumer involvement); participants (description, location, setting, and demographics), intervention (eg, theoretical basis, control/usual care/cointervention, delivery, providers and integrity), outcomes (primary and secondary outcome measures, how assessed and timings of follow-up), and results. Where required, authors will be contacted for clarification or additional information. Completed electronic extraction sheets will be kept as part of the audit trail, should they be required at a later stage to enable data checking.

### Quality Assessment

The evidence and quality of the papers included in the systematic review will be assessed using a recognized tool [43]. A motivation for selecting this tool for quality assessment is that it is suitable for interventions of various study designs, which may be considered for inclusion in this review. As with the data extraction stage, studies will be scored independently by two reviewers, and any disagreements will be resolved through discussion with the other team members.

### Data Synthesis

It is anticipated that there will be several studies that have focused on the effectiveness of mobile phone or tablet apps to facilitate the management of long-term physical conditions in young people. There are expected to be various outcome measures at various time-points for different conditions.

Where there is sufficient homogeneity amongst trials, metaanalyses will be undertaken with the RevMan software as used by the Cochrane Collaboration (RevMan v 4.2.8, Cochrane IMS). Levels of homogeneity can be determined using the chi-squared statistical test. Similar outcome measures will enable a pooled effect size and confidence intervals to be calculated as well as establishing level of publication bias through the use of funnel plots.

Based on the review’s broad inclusion criteria, it is likely that interventions will be implemented at different time points and at various stages of an individual’s illness trajectory. Moreover the population will likely be different age groups living with various physical long-term conditions. To counter this, the following categorizations will be considered for the synthesis: different age categories of young people; interventions developed to facilitate self-management and shared/joint management; whether or not interventions have a theoretical underpinning; short-term and long-term, based on whether any differentiation was made on length of time patient had lived with the condition. Wherever appropriate, pooled estimates will be created and sensitivity analyses will be used to assess the appropriateness of this.

##  Results

This systematic review aims to determine whether mobile phone and tablet apps are effective in young people’s management of physical long-term conditions. Currently, the reviewers are screening papers meeting the search strategy. It is anticipated that by synthesizing included studies, the systematic review results can comment on what components of interventions are most associated with their effectiveness. The completion date for the review is projected to be early-mid-2015.

##  Discussion

### Significance of Findings

Health care advancements mean young people living with long-term physical conditions have improved survival rates [5,44]. However, they have distinct health needs as they transition into adulthood for which regular and appropriate support is paramount [6,45]. While there is evidence suggesting that interventions in the form of mobile phone and tablet apps have great potential [13] and reasonable uptake [25], to our knowledge there has not been a synthesis of studies reporting on their effectiveness in the management of physical long-term health conditions in young people. Therefore, this review will synthesize relevant studies so as to make a definitive statement on the current evidence as well as to illuminate a clear evidence-based direction for future research.

We have an established multidisciplinary team of experts including health care professionals, consumer representatives, and researchers to take this project forward and ultimately develop evidence-based mobile phone and tablet apps for young people with physical long-term conditions. The consumer representatives on the research team have previously undertaken an online survey of 11-19 year olds with Juvenile Idiopathic Arthritis (JIA), which confirmed the need for an app that is codeveloped by people with experience of JIA (personal communication S Stones and S Douglas, 2013). This echoes other reports of young people calling for Web-based interventions to support self-management [46].

A particular strength of this review is that it aims to identify what interventions exist for a variety of long-term conditions (as opposed to condition specific reviews, for example in asthma [38]), which interventions are effective, and with what level of user involvement they were developed and evaluated [47]. In addition to disseminating these findings in a stand-alone review, they will be used as discussion aids in future qualitative studies with young people when developing and evaluating mobile apps. We will adapt the design and methodology of previous work (where members of the current team developed and evaluated an interactive Web app to support parents’ home-based management of their childrens’ long-term conditions [46,47]) to develop an evidence-based app that is effective in meeting the needs and preferences of young people with JIA. By working in collaboration with consumer representatives this app could potentially act as a template, with elements that could be transferred to other conditions. We anticipate our findings will have demonstrable benefit internationally for young people living with physical long-term conditions.

### Conclusions

As yet, the effectiveness of mobile phone or tablet apps to support young people living with long-term conditions is unknown. With the emphasis on limited resources and technology, it is imperative to wholly understand the existing evidence base. This knowledge will serve those considering developing, using or recommending health care apps. Ultimately therefore, this systematic review aims to identify the existing evidence and evaluate the effectiveness of mobile phone and tablet apps for the management of physical long term conditions in young people.

Moreover, by identifying existing evidence and examined current apps, the review’s results will form the first phase of the Medical Research Council (MRC) framework for developing and evaluating complex interventions [48]. The next phase will require the theoretical understanding developed from the review to be supplemented with primary research. We anticipate undertaking focus groups with young people to confirm and further illuminate the findings from the review. Apps identified from the systematic review will be used as discussion aids within the focus groups. Subsequent stages of the MRC framework will focus on designing, developing, and evaluating an app for the management of specific long-term conditions in young people. This will be undertaken in collaboration with young people living with long-term conditions. As well as using the review results as the basis of further research, the research team will disseminate the findings at international conferences and in a prestigious, peer-reviewed journal..

## Multimedia Appendix 1

Example search strategy for Medline Database: Ovid MEDLINE 1946 to January Week 2 2014.
