# Supplementary material for: Mobile Phone and Tablet Apps to Support Young People’s Management of Their Physical Long-Term Conditions: A Systematic Review Protocol
Source: JMIR Res Protoc. 2015 Apr 7;4(2):e40. doi: 10.2196/resprot.4159 (PMC4405621; doi:10.2196/resprot.4159)
Supplement: Supplementary file 1 [file resprot_v4i2e40_app1.pdf]

Multimedia Appendix 1

Example search strategy for Medline Database: Ovid MEDLINE 1946 to January Week 2 2014.

Search History (44 searches)*(close)*

	Searches	**Results**
1	CELLULAR PHONE/	4713
2	COMPUTERS, HANDHELD/	2416
3	((cell$ or mobile$) adj3 phone$).tw.	4162
4	(handheld$ or hand-held$).tw.	6885
5	(smartphone$ or smart-phone$).tw.	888
6	PDA.tw.	5317
7	(personal$ adj3 digital$).tw.	941
8	(windows adj3 (mobile$ or phone$ or cell$)).tw.	83
9	android.tw.	645
10	iOS.tw.	564
11	(Samsung or Nokia or Apple or LG or ZTE or Huawei or TCL or Lenovo or Sony or Yulong or Blackberry or HTC or iphone$ or i-phone$ or ipad$ or i-pad$).tw.	14175
12	(tablet adj3 (device$ or comput$)).tw.	276
13	(mhealth or m-health).tw.	260
14	mobile health.tw.	403
15	m health.tw.	87
16	1 or 2 or 3 or 4 or 5 or 6 or 7 or 8 or 9 or 10 or 11 or 12 or 13 or 14 or 15	35038
17	(child$ or adolescen$ or boy$ or girl$ or teen$ or schoolchild$ or preschool$ or pre-school$ or baby or babies or young person$ or young people or youngster$ or youth$ or juvenile$ or pediatric$ or kid$).tw.	1628475
18	CHILD/ or ADOLESCENT/ or PEDIATRICS/	2304634
19	17 or 18	3043674
20	chronic.tw.	776580
21	(ongoing adj3 (condition or health)).tw.	1004
22	(persistent adj3 (illness$ or disease$)).tw.	4684
23	(long adj term adj3 (illness$ or disease$)).tw.	7836
24	asthma$.tw.	116909

25	arthriti$.tw.	125593
26	diabet$.tw.	395116
27	hypertensi$.tw.	308791
28	cardiovascular$.tw.	266134
29	epilep$.tw.	93981
30	cystic fibrosis.tw.	31933
31	HIV.tw.	225429
32	AIDS.tw.	116704
33	cancer.tw.	1000845
34	haemophili$.tw.	6993
35	haemophili$.tw.	6993
36	sickle cell.tw.	16755
37	coeliac.tw.	6400
38	celiac.tw.	13458
39	thalassemia.tw.	11467
40	cerebral palsy.tw.	14265
41	muscular dystroph$.tw.	17578
42	20 or 21 or 22 or 23 or 24 or 25 or 26 or 27 or 28 or 29 or 30 or 31 or 32 or 33 or 34 or 35 or 36 or 37 or 38 or 39 or 40 or 41	3132566
43	16 and 19 and 42	1148
**44**	**limit 43 to (english language and yr="2003 -Current")**	**754**
